# Mechanisms of auditory masking in marine mammals

**DOI:** 10.1007/s10071-022-01671-z

**Published:** 2022-08-26

**Authors:** Brian K. Branstetter, Jillian M. Sills

**Affiliations:** 1 National Marine Mammal Foundation, 2240 Shelter Island Drive, #204, San Diego, CA 92106 USA; 2 Institute of Marine Sciences, Long Marine Laboratory, University of California Santa Cruz, Santa Cruz, CA 95060 USA

**Keywords:** Masking in marine mammals, Marine mammals and noise, Auditory masking, Informational masking

## Abstract

Anthropogenic noise is an increasing threat to marine mammals that rely on sound for communication, navigation, detecting prey and predators, and finding mates. Auditory masking is one consequence of anthropogenic noise, the study of which is approached from multiple disciplines including field investigations of animal behavior, noise characterization from in-situ recordings, computational modeling of communication space, and hearing experiments conducted in the laboratory. This paper focuses on laboratory hearing experiments applying psychophysical methods, with an emphasis on the mechanisms that govern auditory masking. Topics include tone detection in simple, complex, and natural noise; mechanisms for comodulation masking release and other forms of release from masking; the role of temporal resolution in auditory masking; and energetic vs informational masking.

## Introduction

Auditory masking in marine mammals has garnered attention in recent years due to an increased awareness of the negative effects of anthropogenic noise on hearing. When one sound interferes with a listener’s ability to detect, discriminate, or recognize another sound, auditory masking occurs. The marine environment has always been noisy, because sound travels exceptionally well in ocean environments. Anything that produces sound is a potential noise source (for a review, see Richardson et al. 1995). One type of noise source is non-biological, which includes wind, rain, ice movement, tides, and seismic events. Biological noise comprises any sounds that living organisms produce, such as snapping shrimp snaps, animal vocalizations, and sounds generated by physical interactions with the environment (e.g., fish chewing coral and whales slapping the water's surface). Non-biological and biological sounds have been part of the ocean’s soundscape for millions of years and marine mammals have evolved to be acoustic specialists in this environment. However, there has been a dramatic increase in anthropogenic noise since the industrial revolution (see Richardson et al. 1995; Southall et al. 2019). Noise sources include transportation (e.g., shipping, aircraft, icebreakers), construction (e.g., dredging, pile driving), geophysical surveys (e.g., airgun arrays), military (e.g., sonars, explosives), and ocean science surveys (e.g., seismology, acoustic tomography) (Richardson et al. 1995). Negative impacts from anthropogenic noise can include physical discomfort, pain, or death (e.g., Parsons 2017); permanent and temporary thresholds shifts in hearing sensitivity (e.g., Finneran 2015); increased stress (e.g., Wright et al. 2007; Houser et al. 2020); changes in behavior that may affect individual fitness (e.g., Southall et al. 2009); and auditory masking (for review, see Erbe et al. 2016).

Auditory masking can be defined as “the process by which the threshold of hearing for one sound is raised by the presence of another (masking) sound; and the amount by which the threshold of hearing for one sound is raised by the presence of another (masking) sound, expressed in dB” (American National Standard Institute (ANSI) 1995). The study of auditory masking in marine mammals can be divided into three primary categories, the first being behavioral response studies evaluating whether animals change their behavior to *presumably* mitigate auditory masking (Holt et al. 2009). Anti-masking strategies include, but are not limited to, the Lombard effect (e.g., Holt et al. 2009; Scheifele et al. 2005) and animals re-locating to quieter environments (e.g., Frankel and Clark 2000; Southall 2005) or increasing the number of elements per call to improve detectability (e.g., Turnbull and Terhune 1993; Serrano and Terhune 2001). The second category is based on *in-situ* noise measurements, which typically inform quantitative modeling efforts. These models predict how communication space is reduced relative to a baseline level, often factoring in signal detection capabilities (e.g., Clark et al. 2009). Models may include hearing-related parameters, such as frequency sensitivity, directivity index, and critical ratios, all of which are derived from studying the hearing of animals. The last category, which is the topic of this paper, is the direct study of hearing in noise. This paper will describe the auditory mechanisms that govern masking patterns in marine mammals, reviewing relevant example studies with a focus on psychoacoustical experiments performed in the laboratory.

Psychoacoustics is the study of the relationship between sound and an animal’s mental representation of that sound (Fechner 1860; Fastl and Zwicker 2007). Since mental representations cannot be directly observed, inferences must be made by observing animal behavior. The standard equation describing this relationship is

1 $$\Omega = f\left( S \right),$$

where Ω is the measured behavior, *S* is the physical attribute of sound, and *f(S)* represents the functional relationship between behavior and sound (Yost and Fay 2012). In auditory masking, at least two sounds are present: a signal, which is a sound of interest, and noise, which is a sound interfering with the detection, discrimination, or recognition of the signal. In studies of marine mammal auditory masking, the amplitude of sound is typically represented in decibel (dB) units related to the sound pressure level (SPL) of the stimulus waveform; reference pressures are 1 μPa in water and 20 μPa in air. Common metrics to describe the levels of sound include root-mean-square (dB_RMS_), peak pressure (dB_P_), peak-to-peak pressure (dB_p–p_), pressure spectral density (dB re μPa^2^ / Hz), and 1/3 octave (OTO) band level (for a review, see Au and Hastings 2008). The majority of studies described below measure masked detection thresholds using a go/no-go signal detection task (Stebbins 1970). Animals are presented with a noise background and trained to produce a response (e.g., a paddle press or vocalization) when they hear a signal in the presence of that noise, or to forgo a response if they do not detect a signal. The level of the signal is manipulated using a variety of methods (e.g., adaptive staircase procedure, method of constant stimuli; Stebbins 1970; Levitt 1971) to measure the amplitude at which the animal can detect the signal at a prescribed percent correct (e.g., 50% correct).

## Detection of tones with simple masking noise

### Tone on tone masking

**Fig. 1:**
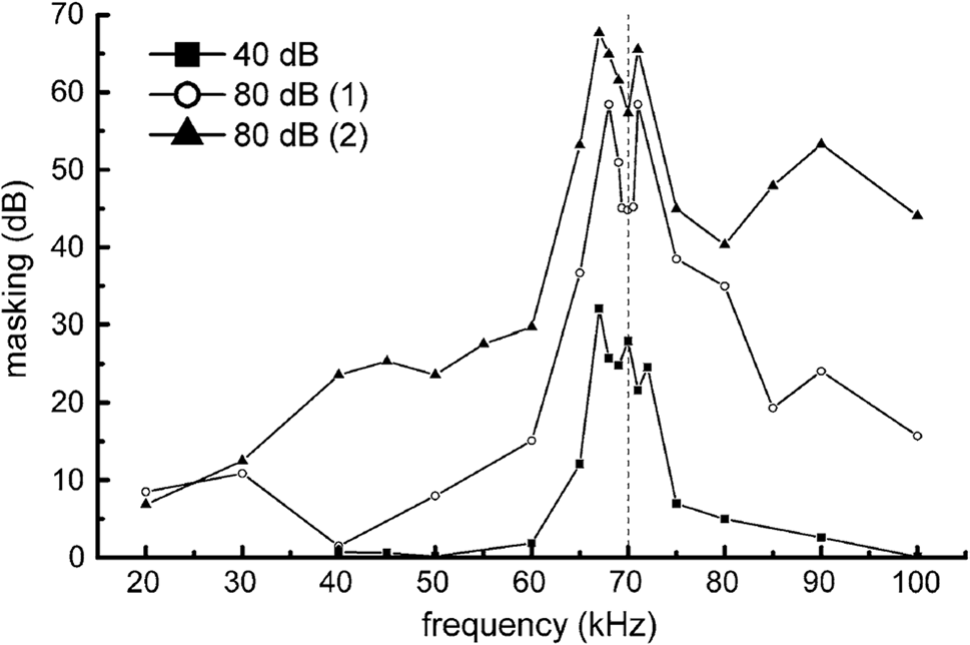
Two-tone masking experiment with a bottlenose dolphin, with masking (dB) shown as a function of frequency (kHz). The vertical dotted line represents the frequency of the masking tone at 70 kHz. Data points represent the dB level above absolute threshold for two levels of the masker: 40 dB and 80 dB re 1 μPa. The 1 and 2 represent two dolphin subjects (adapted from Johnson 1971).

One of the earliest masking experiments to be conducted with a bottlenose dolphin (*Tursiops truncatus*) measured detection thresholds for a pure tone masked by another pure tone (Johnson 1971). In this study, a 70 kHz tone at two different SPLs (40 and 80 dB re 1 μPa) was used as a masking tone. Figure 1 plots how much masking occurred (i.e., the dB level above absolute threshold required to detect the signal at each frequency). Absolute threshold can be defined as the minimum level at which the signal can be detected half of the time without the influence of background noise. The observed masking patterns were consistent with human studies, where higher amplitude tones produced more masking and lower frequencies were better at masking higher frequencies.

Lower frequency tones are better at masking higher frequency tones due to basilar membrane mechanics. Hair cells sensitive to higher frequencies are positioned toward the basal end of the basilar membrane, while lower frequencies with longer wavelengths excite hair cells toward the apical end. Thus, a traveling wave from a low-frequency tone will traverse through and displace the high-frequency portion of the basilar membrane but not vice versa, resulting in the asymmetrical masking pattern.

The largest amount of masking in this experiment occurred when the signal and the masker were similar in frequency. Dips in the masking level near 70 kHz occurred when the signal and noise tones were almost identical in frequency, as a result of the perception of “beats” or amplitude modulation (AM) due to the interaction of the two tones. The AM rate (*M*_*f*_) in Hz is equal to

2 $$M_{f} = \left| {f_{1} - f_{2} } \right|,$$

where *f*_*1*_ and *f*_*2*_ are the frequencies (in Hz) of the signal and noise tones, respectively. Mammalian auditory systems are more sensitive to low-frequency AM rates (Dolphin et al. 1995; Finneran et al. 2007; Viemeister, 1979), so as *M*_*f*_ increases, the perception of beats diminishes and can no longer be used as a cue.

### Tone detection in white noise

Pure-tone signals and white-noise maskers are easy to generate and measure due to their relatively steady state, and are therefore commonly used in psychoacoustic experiments. White noise is a special type of noise where the pressure spectral density is equal or "flat" at each frequency. To digitally generate white noise, the instantaneous amplitude is randomly sampled from a normal or “Gaussian” distribution; hence, the term Gaussian noise is often used interchangeably with white noise.

**Fig. 2:**
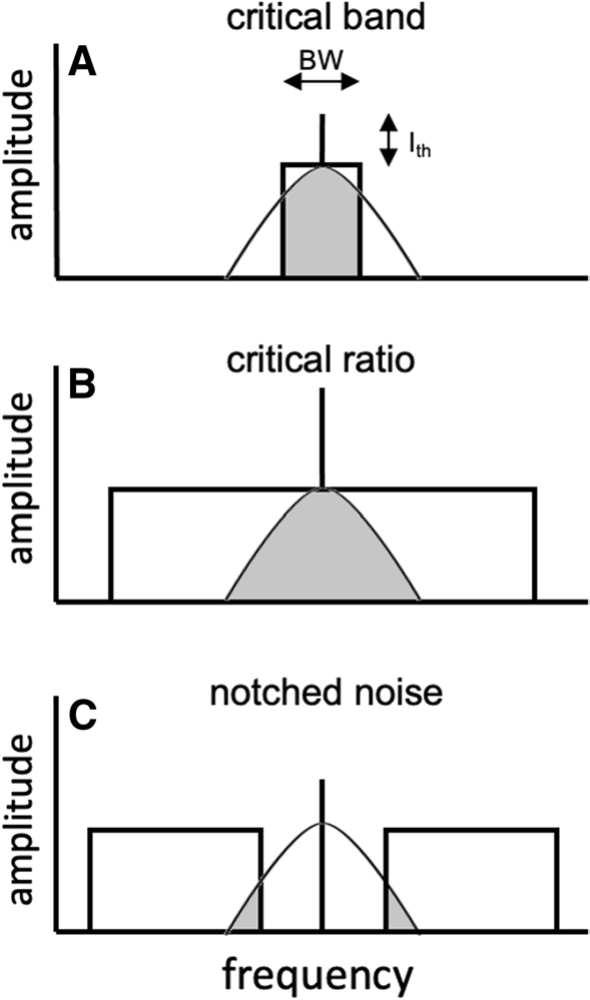
Relationship between (**A**) the critical band, (**B**) the critical ratio, and (**C**) notched-noise masking paradigms. In each panel, the height of the rectangle represents the spectral density level of the masking noise (dB re (1 μPa)^2^ / Hz in water) and the width represents the bandwidth (BW) in Hz. The vertical line (*I*_th_) represents amplitude of the tonal signal at masked threshold (dB re 1 μPa in water). The curve represents a hypothetical auditory filter centered on the signal and the gray portion under the curve represents the amount of noise that contributes to the masking of the signal

In a seminal study with human listeners, Fletcher (1940) discovered that the level of a tonal signal at threshold (*S*_th_) was proportional to the bandwidth of Gaussian noise (Δ*f*) centered on the tone. As the bandwidth increased, detection thresholds increased proportionally, but only up to a *critical bandwidth* (Δ*f*_CB_). Noise frequencies beyond the critical bandwidth had no effect on detection thresholds. Fletcher envisioned a hypothetical band-pass filter or *critical band* centered on the signal, where only noise within the critical band contributed to the masking of the signal (Fig. 2A). The relationship between the signal at threshold (*S*_th_) and the bandwidth of noise within the critical band (Δ*f*_CB_) can be formalized by

3 $$S_{th} = K \cdot N \cdot {\Delta }f_{CB} ,$$

where *N* is the spectral density (in μPa^2^ / Hz) of the noise and *K* is a constant. If *K* is assumed to be equal to one, Eq. 3 can be simplified to

4 $$\Delta f_{CR} = \frac{{S_{{{\text{th}}}} }}{N},$$

where Δ*f*_CR_ is the critical ratio. Rather than performing the relatively time-consuming band-widening procedure necessary for critical band measurements, the auditory filter bandwidth can be estimated by simply measuring the detection threshold of a tonal signal in broadband noise to calculate the critical ratio (Fig. 2B). The term *broadband noise* is, within this paper, operationally defined as a bandwidth greater than the auditory filter bandwidth.

For humans with a relatively narrow range of hearing (approximately 20 Hz to 20 kHz; Yost and Fay 2012), critical ratios can be estimated using a noise bandwidth that encompasses the full range of hearing. For marine mammals, some of which have an extremely broad hearing range (e.g., < 75 Hz to > 150 kHz for the bottlenose dolphin; Johnson 1967), critical ratios are often measured with noise bands that are one-third octave wide or greater (e.g., Au and Moore 1990; Branstetter et al. 2017, 2021). If the level of the signal (*S*_th_) is expressed in terms of SPL and the level of the noise (*N*) is expressed as the pressure spectral density, the critical ratio (CR) can be calculated by

5 $${\text{CR }} = \, S_{{{\text{th}}}} {-} \, N.$$

**Fig. 3:**
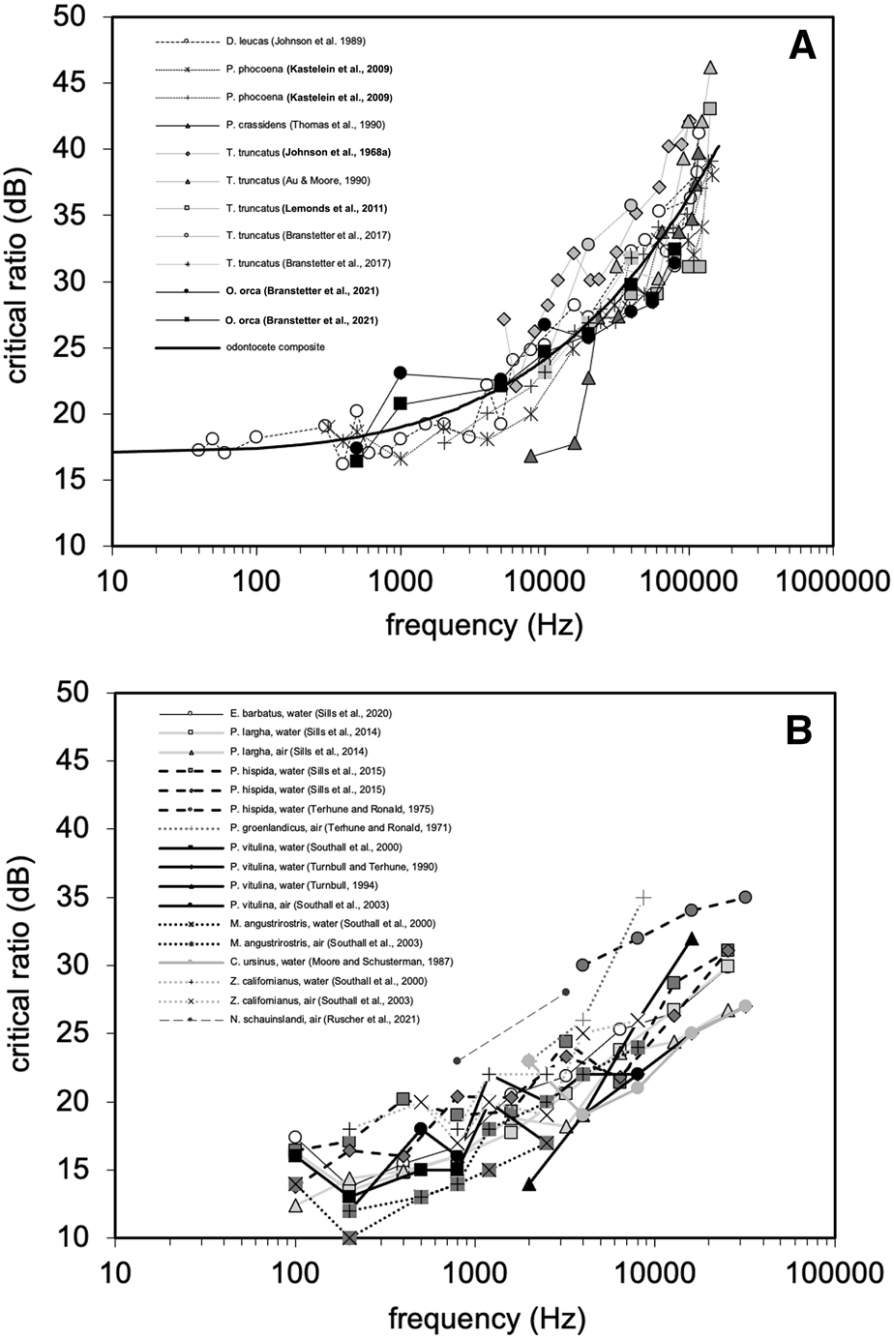
Critical ratios for odontocetes (**A**) and pinnipeds (**B**) as a function of frequency. The curve in panel **A** represents a model fit to the cumulative data. For pinniped critical ratios in panel **B**, the legend indicates the species and the sound medium (air or water) in which the critical ratios were measured. (Panel **A** was adapted from Branstetter et al. 2021; Finneran and Branstetter 2013)

Critical ratios have been measured for five odontocete species: *Delphinapterus leucas* (Johnson et al. 1989), *Orcinus orca* (Branstetter et al. 2021), *Phocoena phocoena* (Kastelein and Wensveen, 2008; Kastelein et al. 2009), *Pseudorca crassidens* (Thomas et al. 1990), and *Tursiops truncatus* (Au and Moore 1990; Branstetter et al. 2017; Johnson 1968a; Lemonds et al. 2011). Auditory masking (at least energetic masking) is thought to occur at the mechano-transduction level of the cochlea (Recio-Spinoso and Cooper 2013). Clear differences in basilar membrane size and morphology exist within odontocetes (Ketten 1992a); however, despite large differences in ear morphology (Ketten 1992b), functional head size (Heffner and Heffner 2008), and frequency sensitivity (see NOAA Fisheries 2018), critical ratios for toothed whales species are remarkably similar (Fig. 3A). Among pinnipeds, critical ratio measurements are available for nine species: *Callorhinus ursinus* (Moore and Schusterman, 1987), *Erignathus barbatus* (Sills et al. 2020), *Mirounga angustirostris* (Southall et al. 2000, 2003), *Neomonachus schauinslandi* (Ruscher et al. 2021), *Pagophilus groenlandicus* (Terhune and Ronald 1971), *Phoca largha* (Sills et al. 2014), *Phoca vitulina* (Renouf 1980; Southall et al. 2000, 2003; Terhune 1991; Turnbull 1994; Turnbull and Terhune 1990, 1993)*, Pusa hispida* (Sills et al. 2015; Terhune and Ronald 1975), and *Zalophus californianus* (Southall et al. 2000; 2003). Critical ratios measured for seals and sea lions (Fig. 3B) are consistently low across a wide range of frequencies relative to many terrestrial mammals (Fay 1988). This suggests that the auditory systems of these marine mammals possess a refined ability to detect signals within background noise.

A feature of mammalian auditory filters is that bandwidth typically increases as a function of the center frequency of the filter, represented by

6 $$Q \, = \, f_{o} /\triangle\ f,$$

where *f*_*o*_ is the center frequency of the filter, Δ*f* is the filter bandwidth, and *Q* is a quality factor. As a result of this relationship, the bandwidth of noise that contributes to the masking of a signal and the amount by which the signal must exceed that noise to be detected are both greater at higher frequencies. This is reflected by a general increase in both critical bands and critical ratios as a function of frequency (Fig. 3).

Fletcher’s auditory filter model (Fletcher 1940) has developed into what is now referred to as the power spectrum model (PSM) of masking (Moore 1993). The PSM makes the following assumptions:

1. The auditory system can be modeled as a bank of continuously overlapping band-pass filters.
2. Only the spectral components within an auditory filter contribute to the masking of a signal centered within that filter.
3. Signal detection occurs by monitoring an energy detector at the output of the filter. A signal plus noise interval will result in an increase in filter output compared to a noise alone interval.
4. Signal threshold is proportional to the spectral density of noise within a filter centered on the signal. Noise is represented by its long-term spectra.

The model can be formalized by

7 $$P_{s} = K\mathop \smallint \limits_{0}^{\infty } N\left( f \right)W\left( f \right)df,$$

where *P*_*S*_ is the power of the signal, *N(f)* is the noise power spectrum, and *W(f)* is the auditory filter function, or the shape of the auditory filter. Auditory filter shapes have been measured behaviorally for two odontocete species (Finneran et al. 2002; Lemonds 1999; Lemonds et al. 2011) using a notched-noise masking paradigm (Fig. 2C). This involves measuring the threshold of a sinusoidal signal as a function of the width of a spectral notch in masking noise around the signal frequency. The masking data can then be fit to a two-parameter rounded exponential function (Patterson 1976; Patterson et al. 1982) or *roex* function

8 $$W(g) = (1 - r)(1 + pg)e^{( - pg)} + r,$$

where *g* is the normalized frequency deviation from the signal frequency

9 $$g = \frac{{\left| {f - f_{o} } \right|}}{{f_{o} }}.$$

**Fig. 4:**
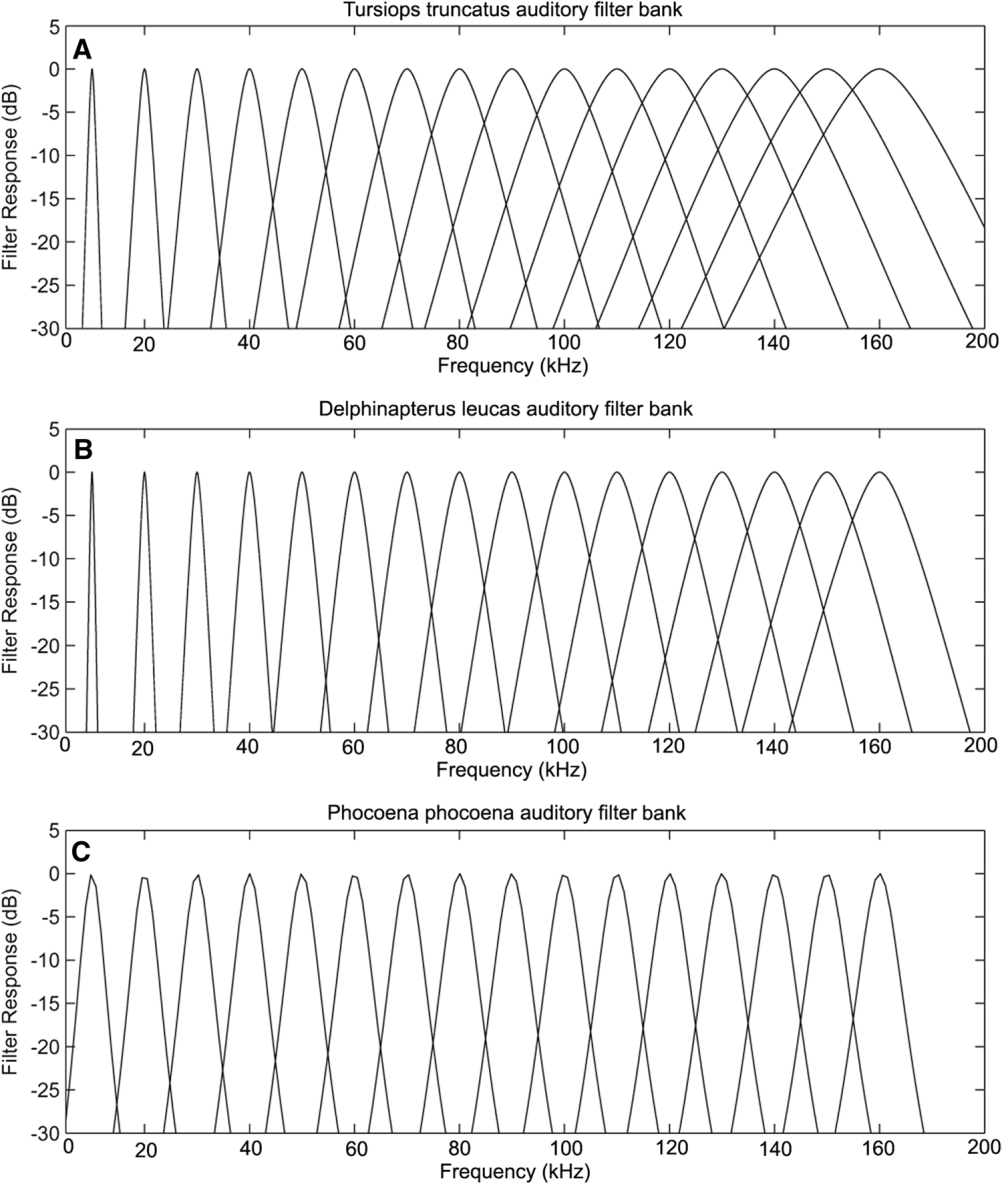
Auditory filter banks for (**A**) *Tursiops truncatus* (**B**), *Delphinapterus Leucas*, and (**C**) *Phocoena phocoena*. (Adapted from Finneran and Branstetter 2013; Au et al. 2009)

Here, *f* is the cut-off frequency of the notched noise in Hz and *f*_*o*_ is the center frequency of the notch, and *p* and *r* are fitting parameters. The roex filter is defined in the frequency domain, but lacks impulse response timing and phase characteristics found in physiological studies (Lyon et al. 2010). Conversely, the gammatone filter is virtually identical to the roex filter in the frequency domain, but is defined in the time-domain; it is often used for modeling purposes (Branstetter et al. 2020; Lyon et al. 2010; Roitblat et al. 1996). Figure 4 displays gammatone auditory filter banks derived from roex filters (Branstetter et al. 2007; Roitblat et al. 1996) for a bottlenose dolphin (Lemonds 1999), a beluga whale (*Delphinapterus leucas*; Finneran et al. 2002), and a harbor porpoise (*Phocoena phocoena*; Popov et al. 2006). The gammatone filter bank models can be used in computational simulations of the hearing system to help describe the discrimination and classification capabilities of odontocetes (Branstetter et al. 2007, 2020; Roitblat 1998; Au et al. 2009), but have yet to be used in simulations of auditory masking.

When predicting auditory masking for mitigation purposes, critical ratios have become a standard metric used (e.g., Clark et al. 2009; Erbe et al. 2016)—along with the PSM of masking—due to their relative ease of data collection and model simplicity. For example, the odontocete critical ratio at 10 kHz is about 24 dB (Fig. 3A). If broadband ocean noise was recorded with a spectral density level of 100 dB re (1 μPa)^2^ /Hz @ 10 kHz, a received 10 kHz signal would need to be at least 124 dB re 1 μPa to be detected by most odontocetes. Although the critical ratio and PSM are simple and useful, the spectral–temporal complexity of many biological signals and ocean noise sources can lead to both masking release (MR) and elevated levels of masking, which will be discussed in subsequent sections.

## Signal detection with complex sounds

### Amplitude modulation, temporal resolution, and multiple looks

**Fig. 5:**
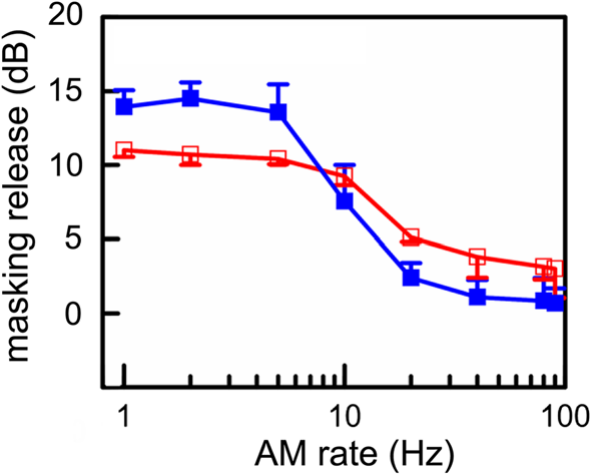
Masking release (dB) as a function of AM rate with SAM maskers and a 4 kHz tonal signal. The two colors represent two different harbor porpoise participants (Adapted from Kastelein et al. 2021)

Many sounds in nature will have a spectral–temporal pattern more complex than Gaussian noise (Nelken et al. 1999). Thus, masking patterns derived from Gaussian noise may be considered a special case. One of the most common features of natural ocean noise is amplitude modulation caused by a reverberant environment, or the animals themselves. Kastelein et al. (2021) measured the harbor porpoise’s ability to detect a 4 kHz tone embedded in sinusoidal amplitude modulated (SAM) noise. A release from masking up to 14 dB relative to Gaussian noise resulted for AM rates between 1 and 5 Hz. From 5 to 20 Hz, MR gradually decreased to levels similar to masking with Gaussian noise (Fig. 5). The results are consistent with a within-valley or a “dip” listening model (Buus 1985) where listening occurs in the troughs of the amplitude modulated noise where the signal-to-noise ratio is highest. As the modulation rate increases, the temporal resolution of the animal’s hearing system will begin to “smear” the temporal envelope of the noise, perceptually reducing the troughs in the noise, thus increasing tone detection thresholds.

In humans, temporal resolution of SAM noise can be described by the temporal modulation transfer function (TMTF), which has a low-pass shape. The ability to discriminate between Gaussian and SAM noise decreases rapidly above 100 Hz (Viemeister 1979). With higher AM rates and lower AM depths, SAM noise and Gaussian noise become indistinguishable. In odontocetes, temporal resolution is complex, and there appears to be at least two distinct mechanisms: one for transient sonar signals (Johnson et al. 1988; Moore et al. 1984) and another for longer duration signals (Johnson 1968b). For broadband transient signals similar to biosonar clicks, temporal resolution has been measured with an integration time of approximately 264 μs (Johnson et al. 1988; Moore et al. 1984). However, for tonal signals, integration times are frequency dependent and on the order of tens to hundreds of milliseconds. For example, the harbor porpoise has an integration time of approximately 277 ms at 4 kHz (Kastelein et al. 2021). If a 277 ms integration window were applied to amplitude modulated noise, AM rates above 4 Hz (4 Hz period = 250 ms) would likely be smeared, suggesting that different mechanisms govern the integration of tonal signals and the resolution of AM noise. Kastelein et al. also demonstrated that signals with longer durations (i.e., 1000 and 2000 ms) were easier to detect than a 500 ms signal, despite the fact that all three exceed the 277 ms integration time. The longer duration signals provide more opportunities for detecting the signal, which is consistent with a statistical “multiple looks” model (Viemeister and Wakefield 1991).

Currently, there is no consistent or single model of temporal integration for odontocetes. The idea that the odontocete auditory system analyses sound on different timescales (i.e., one for sonar-like signals and one for long-duration signals) is not without precedence. Evidence suggest that songbirds may have two distinct temporal integration times, one for short-duration signals between 10 and 30 ms, and another for longer duration signals between 500 and 700 ms (Narayan et al. 2006). Human speech may even be processed on a dual scale (Teng et al. 2016). How temporal integration affects auditory masking in marine mammals is a topic in need of investigation and will be revisited below in the section on comodulation masking release.

**Fig. 6:**
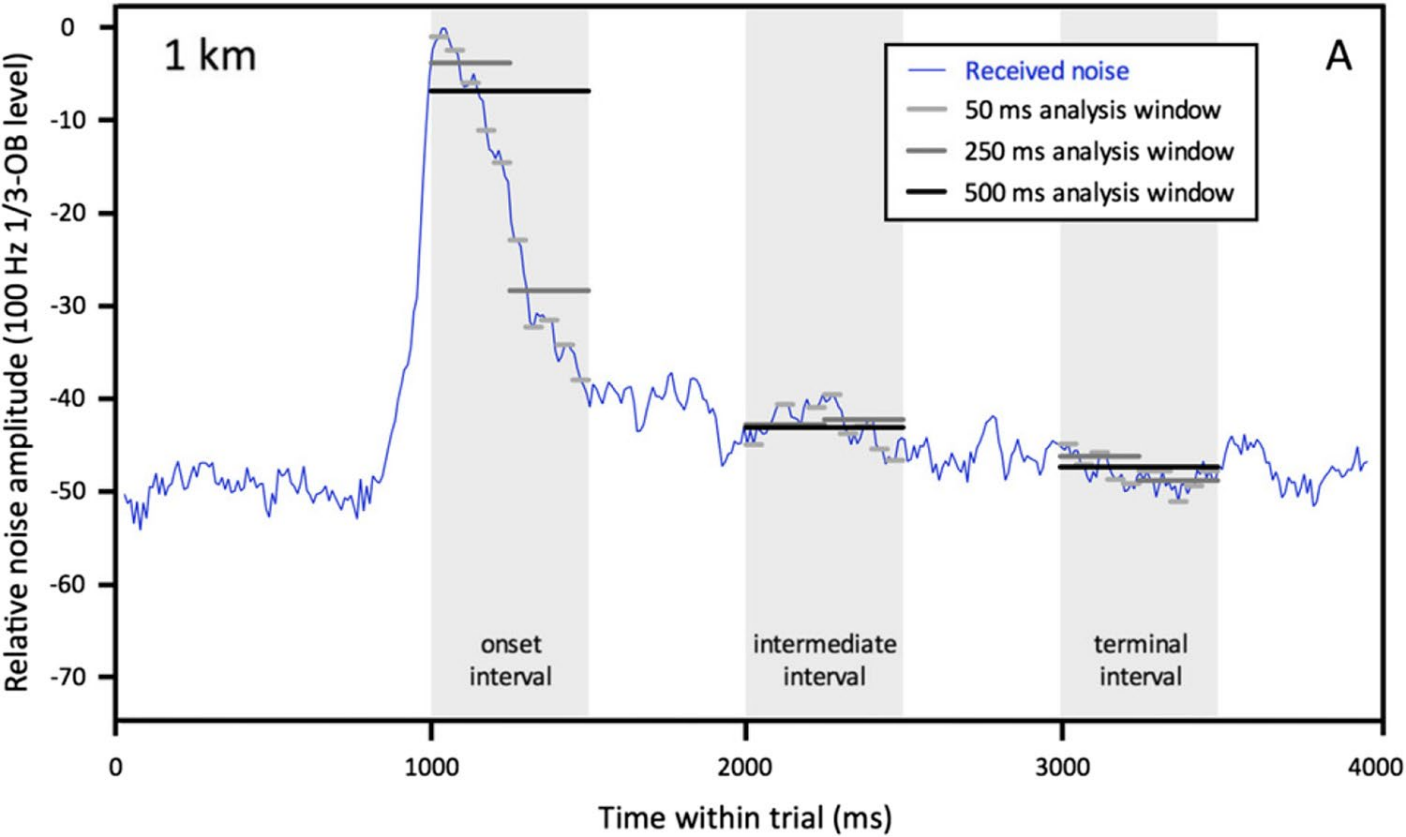
Example of time-window analysis. Noise levels were calculated over different window durations between 50 and 500 ms. The levels were used to predict auditory masking when a 500 ms signal occurred either in the onset, intermediate, or terminal interval of the seismic airgun noise. (Adapted from Sills et al. 2017)

Sills et al. (2017) measured detection thresholds for a spotted seal (*Phoca largha*) and a ringed seal (*Pusa hispida*) listening for tonal signals in seismic airgun noise. Seismic airgun noise is an impulsive sound produced by a rapid release of compressed air (Vaage et al. 1983). Airgun arrays typically have a 10 s duty cycle, with an interval between pulses during which noise may or may not return to background levels (e.g., Guan et al. 2015). Sills et al. applied a time-window analysis, where windows of varying duration were used to measure the signal-to-noise ratio. The results indicate that the animals were likely using a “dip-listening” strategy. Detection thresholds were well predicted by critical ratios applied to short-duration temporal windows, particularly when noise amplitude fluctuated substantially over the duration of the signal. When noise levels were less variable, as in the ‘intermediate’ and ‘terminal’ intervals farther from pulse onset, analyses across longer temporal windows were also good predictors of masking (Fig. 6).

### Comodulation masking release

**Fig. 7:**
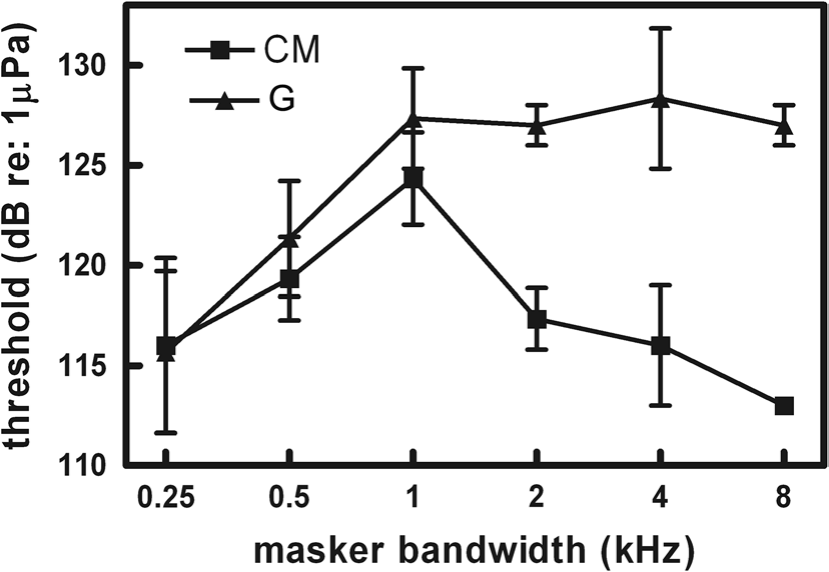
Comparison between masking patterns from comodulated (CM) noise and Gaussian (G) noise for one bottlenose dolphin. Masked thresholds at 10 kHz are shown (with standard deviation) as a function of masker bandwidth. (Adapted from Branstetter and Finneran 2008)

Many natural sounds are both broadband and amplitude modulated (Nelken et al. 1999). When the amplitude modulation is coherent across frequency regions (i.e., multiple auditory filters), the noise is called comodulated and can result in comodulation masking release (CMR) (Branstetter and Finneran 2008; Buschermohle et al. 2007; Hall and Grose 1988). Animal auditory systems appear to use comodulation to group and separate sounds in complex auditory scenes (Klump and Nieder 2001; Nelken et al. 1999). In a band-widening experiment, Branstetter and Finneran (2008) compared detection thresholds of a 10 kHz tone masked by Gaussian noise (G) and by comodulated noise (CM), which was produced by multiplying Gaussian noise by low-pass noise. When the bandwidths of G and CM noise were narrower than the width of an auditory filter, thresholds increased as a function of bandwidth in a manner consistent with the PSM. However, when bandwidth exceeded the width of an auditory filter (i.e., it was processed by more than one auditory filter), thresholds for tones within CM noise decreased as a function of noise bandwidth. The effect was pronounced, with a noise bandwidth of 8 kHz resulting in substantial release from masking relative to Gaussian noise of the same bandwidth and pressure spectral density (Fig. 7). In other words, more total noise energy resulted in less masking, a result that is not intuitive and is at odds with the PSM. The breakpoints for both G and CM noise at a bandwidth of 1 kHz (Fig. 7), which is the bandwidth of the auditory filter centered on the 10 kHz tone, suggest that a processing transition occurs. For Gaussian noise, spectral frequencies beyond the auditory filter no longer contribute to masking of the signal (i.e., the PSM holds). However, CM noise beyond the auditory filter appears to enhance the detectability of the signal.

**Fig. 8:**
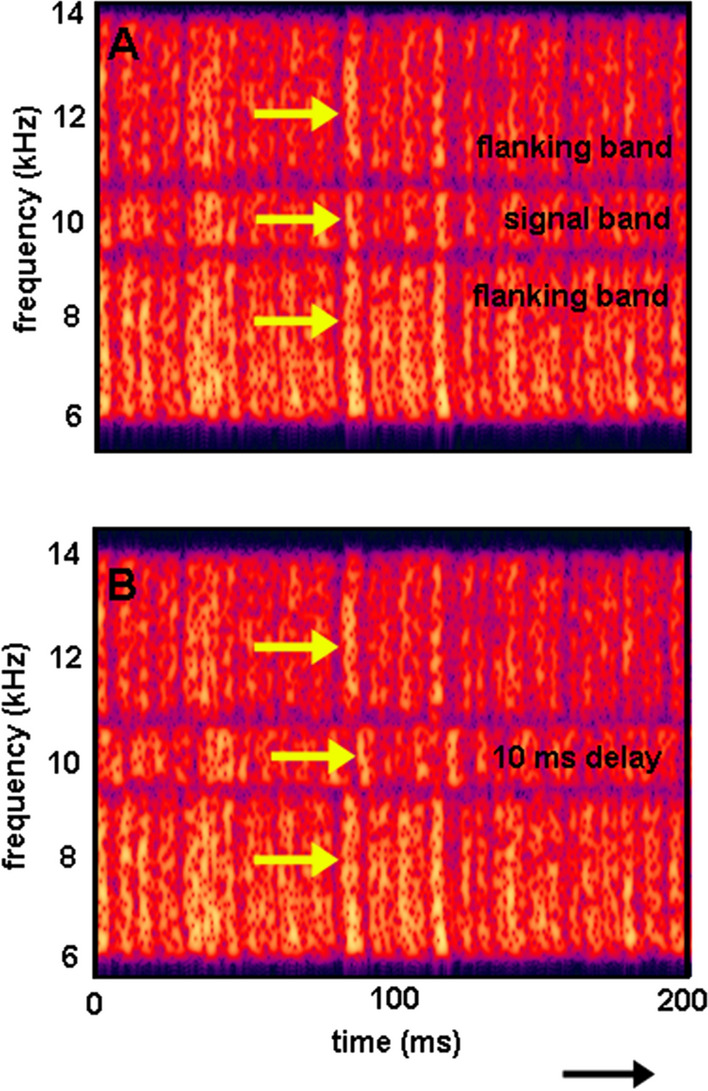
Spectrograms of masking noise with a signal band and two flanking bands. The signal band in Panel B is delayed by 10 ms, which decorrelates the envelopes. (Adapted from Branstetter et al. 2013a)

**Fig. 9:**
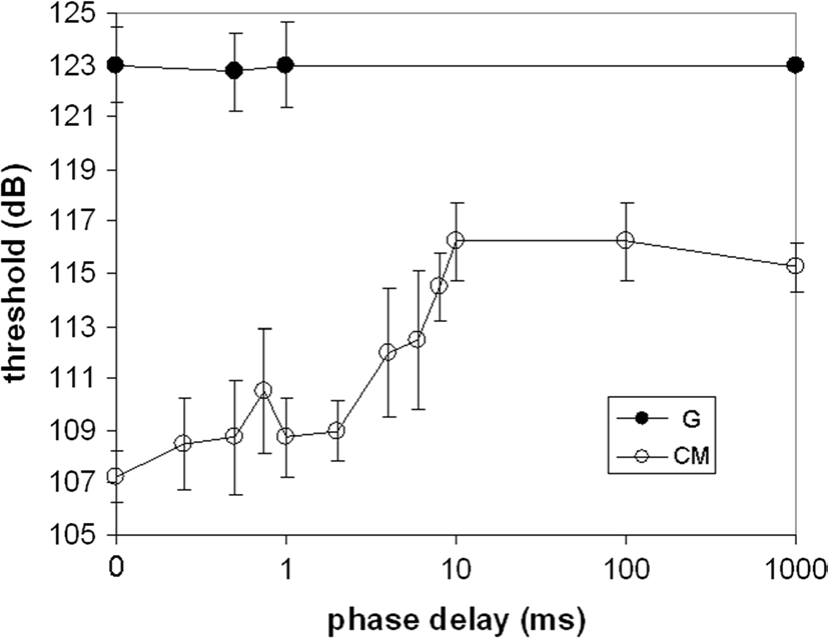
Detection thresholds in Gaussian (G) and comodulated (CM) noise as a function of the phase delay of the signal band, as shown in Fig. 8. The phase delay had no effect on thresholds measured in the presence of G noise. However, for CM noise, the phase delay accounted for approximately 7 dB of masking release and AM depth accounted for approximately 6 dB of masking release. Error bars represent standard deviations. (Adapted from Branstetter et al. 2013a)

To test the effects of both amplitude modulation and the degree of coherence across frequency regions, an additional experiment was conducted (Branstetter et al. 2013a). Masking noise was divided into a signal band, which was the width of an auditory filter and centered on a 10 kHz signal, and two flanking bands (see Fig. 8). The signal band was then progressively delayed relative to the flanking bands, thus decreasing the level of across-channel coherence. This procedure was repeated for both G and CM noise. For the G noise, the delay had no effect on detection thresholds, since Gaussian noise is not coherent. However, for CM noise, the delay disrupted the CMR effect, resulting in increasing thresholds for delays between 2 and 10 ms (Fig. 9). The increase in thresholds as a function of delay was approximately 0.85 dB/ms delay (Branstetter et al. 2013a). Although CMR was disrupted by decorrelating the envelopes, the effect was only about 7 dB of masking release. This suggested an additional mechanism accounted for the remaining CMR effect, namely “dip” listening. This hypothesis was tested by adjusting the depth of modulation of CM noise from 100 to 0% modulated. When AM depth decreased from 100 to 90%, thresholds increased by 6 dB and then remained stable from 90 to 0% (Branstetter et al. 2013a). These results provided evidence that the total CMR effect was the combination of two factors, across channel coherence (about 7 dB of MR) and dip-listening (about 6 dB of MR).

**Fig. 10:**
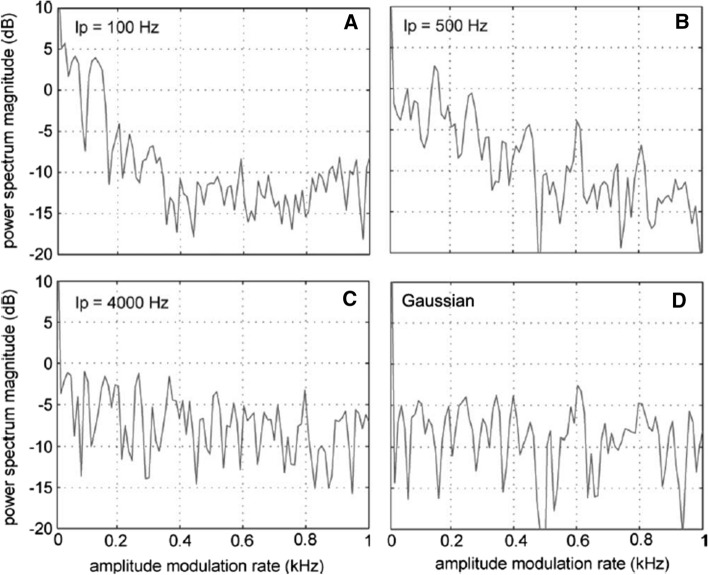
Power spectra from envelopes of different comodulated (CM) noise. Panels **A**, **B**, and **C** show comodulated noise produced by multiplying Gaussian noise with low-pass noise where the low-pass cutoff was 100, 500, and 4000 Hz, respectively. Panel **D** is from Gaussian noise. (Adapted from Branstetter and Finneran, 2008)

**Fig. 11:**
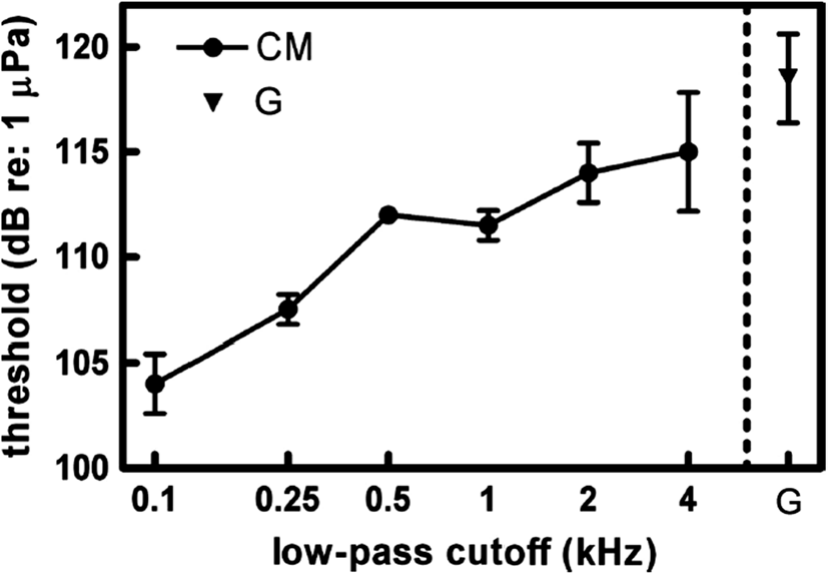
Detection thresholds in comodulated (CM) noise compared to Gaussian (G) noise. CM noise is generated by multiplying G noise by low-pass noise. Detection thresholds increase as a function of the low-pass filter cutoff. (Adapted from Branstetter and Finneran 2008)

Temporal resolution plays a central role in CMR and has been shown to be related to the TMTF (Berg 1996). As mentioned above, CM noise can be synthesized by multiplying low-pass noise by Gaussian noise. The cut-off frequency of the low-pass filter affects the AM rates of CM noise (Fig. 10), which provides a mechanism for evaluating the effect of AM rate on detection thresholds. In an experiment to this effect, Branstetter and Finneran (2008) found that lower AM rates—associated with lower-frequency cutoffs—resulted in a greater amount of CMR (Fig. 11). The same pattern was displayed in an AM masking study by Kastelein et al. (2021); however, at very different AM rates. In Kastelein et al. (2021), MR did not occur for AM rates above 20 Hz. In Branstetter and Finneran (2008), the CMR effect was still noticeable with AM rates up to at least 500 Hz (Fig. 11). The source of the variability between the two studies is unknown; however, a bottlenose dolphin was the subject in Branstetter and Finneran (2008), while a harbor porpoise was the subject in Kastelein et al. (2021), suggesting species-specific differences. More studies are required to determine the relationship between temporal resolution and masking release.

### Masking with environmental noise

**Fig. 12:**
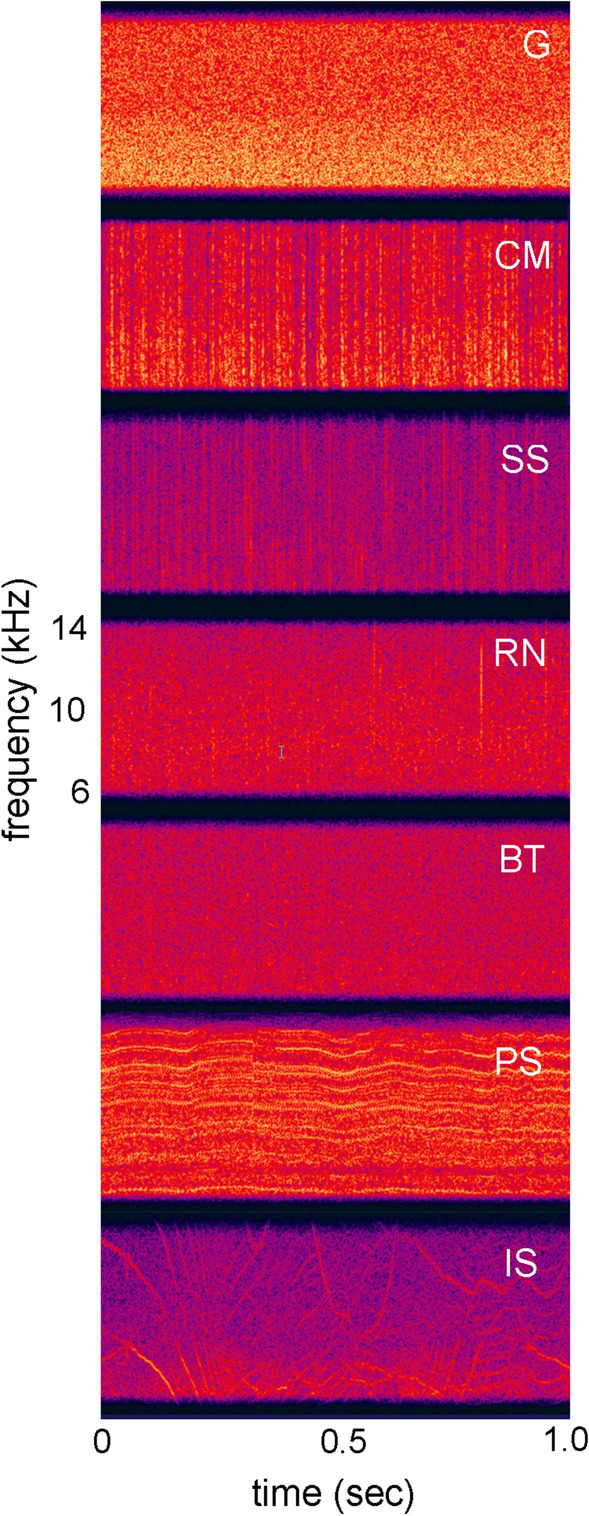
Spectrograms of different noise types: Gaussian (G), comodulated (CM) noise, snapping shrimp (SS), rain (RN), boat noise (BT), a pile saw (PS), and ice squeaks (IS). All noise types had flat spectral densities of 95 dB re (1 μPa)^2^/Hz. (Adapted from Branstetter et al. 2013a)

**Fig. 13:**
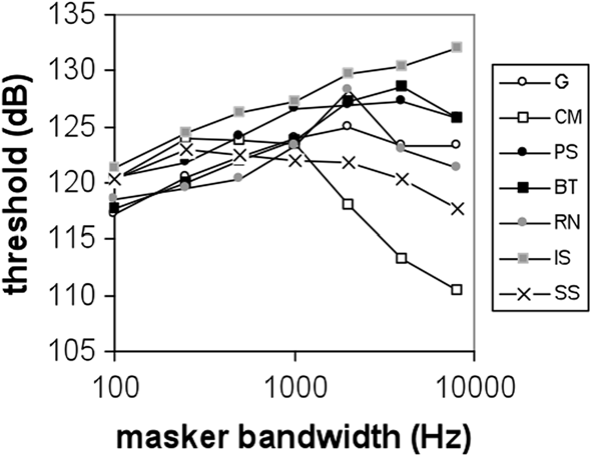
Masking patterns for a 10 kHz tone in the presence of different noise types. All noise types display similar masking patterns up to a bandwidth of 1 kHz. For noise bandwidths greater than 1 kHz, masking patterns diverge, representing different processing mechanisms. Noise types are Gaussian (G), comodulated (CM) noise, snapping shrimp (SS), rain (RN), boat noise (BT), a pile saw (PS), and ice squeaks (IS). (Adapted from Branstetter et al. 2013a)

The spectral-temporal properties of environmental noise can be varied and complex (Erbe et al. 2016), and as a result, lead to different levels of auditory masking. Comodulation masking release has been observed with different environmental noise types. For example, a beluga whale was trained to detect conspecific vocalizations within four different noise types: G noise, ice-creaking noise, propeller cavitation, and a bubbler noise system (Erbe and Farmer 1998, 2000). There was an 11-dB spread in masked detection thresholds, despite equal spectral densities between the noise types. Both dip-listening and CMR were likely candidates for the observed masking release. In a related experiment, two bottlenose dolphins demonstrated a release from masking with “environmental noise,” which was a recording of ambient noise in San Diego Bay (Trickey et al. 2011). The recording was dominated by snapping shrimp and resulted in a 6-dB release from masking compared to G noise of the same spectral density. In a band-widening experiment with a bottlenose dolphin (Branstetter et al. 2013a), detection thresholds were measured for a 10 kHz tone masked by seven noise types: G noise, CM noise, snapping shrimp (SS), rain (RN), boat noise (BT), a pile saw (PS), and ice squeaks (IS). Spectrograms of each noise type are displayed in Fig. 12. For narrow-band maskers centered on 10 kHz (i.e., 1 kHz bandwidth and below), all noise types had a masking pattern consistent with the PSM, in which detection thresholds increased as a function of bandwidth (Fig. 13). However, when bandwidth exceeded the width of the auditory filter, masking patterns diverged. Two noise types, SS and CM, resulted in a release from masking, while RN and G produced a masking pattern consistent with the PSM. Interestingly, both PS and IS noise, which have strong frequency-modulated components, resulted in elevated masking thresholds. To a human listener, both PS and IS have tonal qualities, making them categorically similar to the tonal signal. Informational masking may have been responsible for these elevated thresholds, which will be discussed below.

**Fig. 14:**
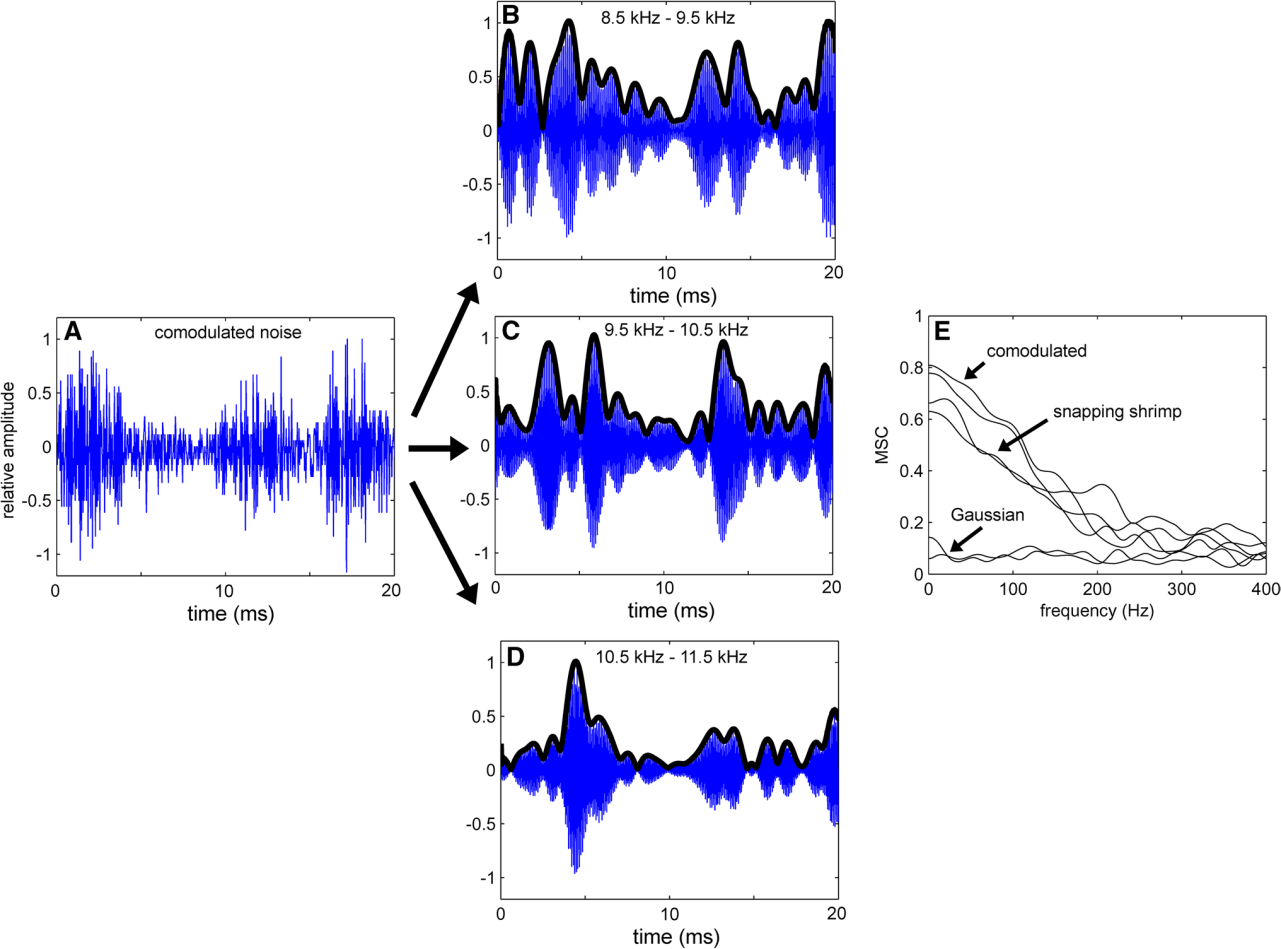
Processing stages used to calculate the comodulation index. Panel A is the time-domain waveform. Panels B, C, and D represent the outputs of the band-pass-filtered waveform. Thick black lines represent the Hilbert envelope. Panel E represents the magnitude squared coherence (MSC) between the temporal envelope of the signal band and the flanking bands for three different noise types. (Adapted from Branstetter et al. 2013b)

**Fig. 15:**
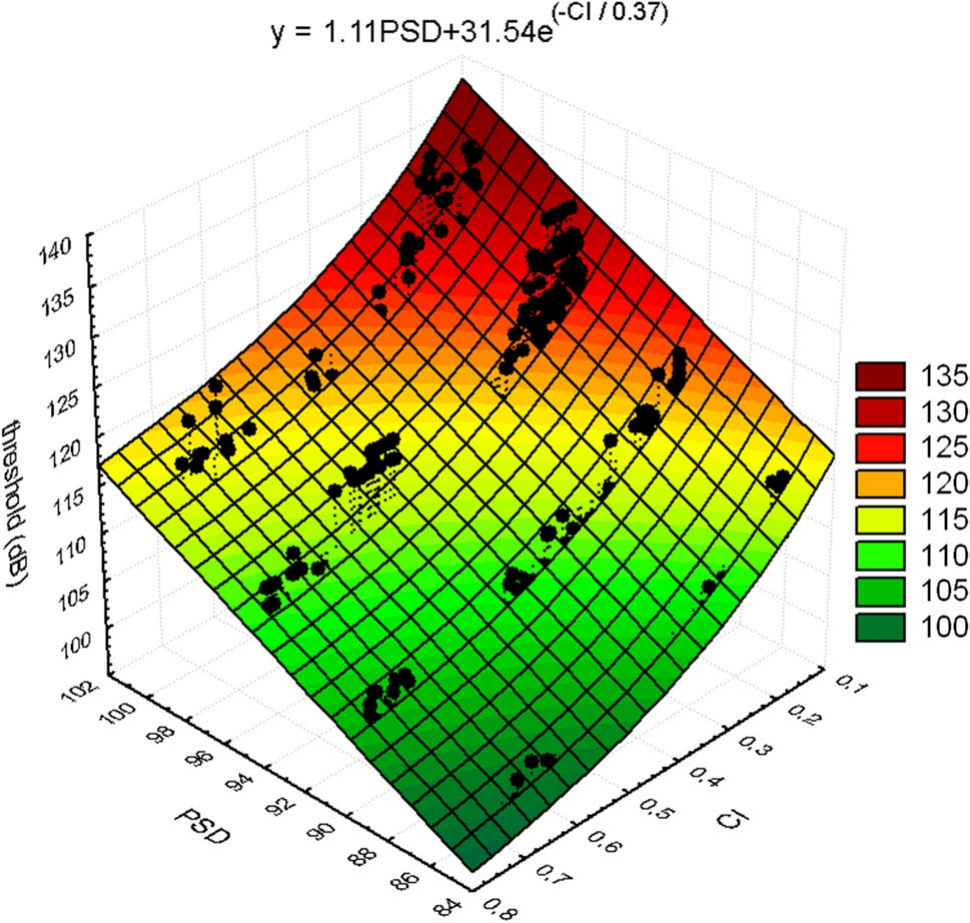
Surface plot of the exponential model used to describe dolphin detection thresholds in 12 different noise types with different levels of masking. PSD is the pressure spectral density and CI is the comodulation index. (Adapted from Branstetter et al. 2013b)

Although a mechanistic model has not been applied to describe and predict auditory masking in marine mammals with different noise types, Branstetter et al (2013b) produced a linear model to describe masking patterns with 12 different noise types and detection thresholds from three different bottlenose dolphins. Predictor variables were noise statistics, and they were divided into three categories: (1) waveform metrics (e.g., RMS and kurtosis), (2) frequency spectrum metrics (e.g., spectral density), and (3) temporal envelope metrics (e.g., envelope kurtosis, comodulation index). The comodulation index (CI) is a measure of how correlated temporal envelopes are across auditory filters. CI is calculated by band-pass filtering the noise sample into three bands that simulate auditory filters. The three bands are a signal band that is centered on the signal, and two flanking bands. The Hilbert envelope env*(t)* is then calculated from the output of each filter

10 $${\text{env}}(t) = \sqrt {f^{2} (t) + h^{2} (t)} ,$$

where *f(t)* is the time-domain waveform and *h(t)* is the Hilbert transform. The magnitude squared coherence is then calculated between the Hilbert envelopes of the signal band and each flanking band. The process is displayed in Fig. 14. An exponential decay model produced the best fit to the cumulative masking data

11 $$y = b_{1} PSD + b_{2} e^{{( - CI/b_{3} )}} ,$$

where *y* is the predicted detection threshold, PSD is the pressure spectral density, and *b*_1_, *b*_2_, and *b*_3_ are fitting parameters. Values for *b*_1_, *b*_2_, and *b*_3_ were 1.11, 31.54, and 0.37, respectively. A surface plot of the model can be found in Fig. 15. The PSD factor is linear, which is consistent with the PSM of masking: for every dB increase in the spectral density, there is a 1.11 dB increase in the detection threshold. The detection threshold is then mediated by the exponential decay function associated with the value of the CI (Fig. 15).

### Detection of complex signals in noise

The ability of the critical ratio to predict auditory masking levels is limited with non-Gaussian noise (e.g., Branstetter et al. 2013a, b). Similarly, complex signals can result in a departure from critical ratio predictions. Although the majority of auditory masking experiments have employed pure-tone signals, pure-tone sounds in nature are rare. Sounds that are perceived to have a tonal quality, such as a dolphin whistle, invariably contain multiple harmonics (Lammers et al. 2003), amplitude modulation (Jones et al. 2022), and frequency modulation (Janik and Sayigh 2013).

**Fig. 16:**
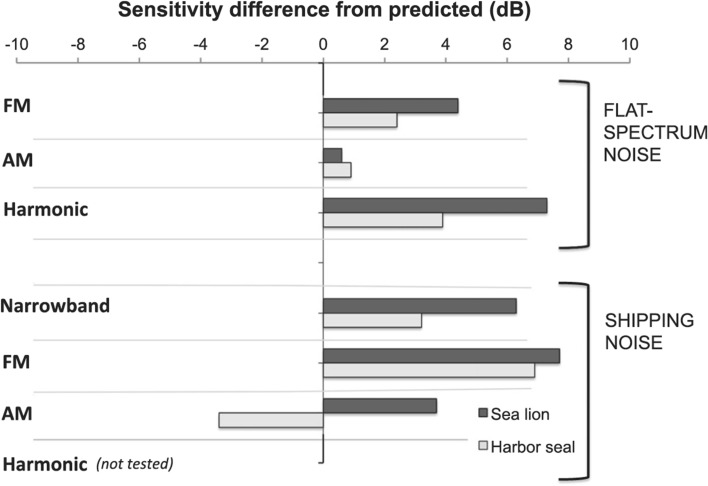
Sensitivity difference from predicted (dB) for a harbor seal and a California sea lion listening for complex signals in the presence of flat-spectrum (Gaussian) noise or shipping noise. Positive values indicate that measured sensitivity was greater than predicted by critical ratios and a power spectrum model of masking. (Adapted from Cunningham et al. 2014)

Cunningham et al. (2014) tested whether critical ratio predictions would generalize to complex signals. Masked detection thresholds were measured for a California sea lion (*Zalophus californianus*) and a harbor seal (*Phoca vitulina*), where the signals were either AM, frequency-modulated (FM), or contained multiple harmonics. For Gaussian-noise maskers, all signal types resulted in enhanced detectability compared to pure-tone signals (Fig. 16). For a shipping noise masker, which was AM and CM, the FM signal again resulted in enhanced detectability for both subjects. Conversely, for the harbor seal listener, detection of the AM signal in shipping noise resulted in more masking than expected (Fig. 16). The similarity between the AM signal and AM shipping noise may be an example of informational masking, which is covered below.

## Spatial release from masking

Current models of auditory masking (e.g., critical ratios and the power spectrum model) assume that both signal and noise are emitted from the same location, resulting in worst-case-scenario masking (Clark et al. 2009). In real ocean environments, noise sources are typically spatially separated from biologically relevant signals. Under such conditions, spatial release from masking (SRM) can occur (e.g., Au and Moore, 1984; Holt and Schusterman, 2007; Popov et al. 2020). In humans, where research on this topic is extensive, the position of sound sources relative to a listener can act as one of the most salient cues to segregate multiple sounds in a complex auditory scene (Bregman 1990). Marine mammals have excellent sound localization abilities and directional receiving beam patterns (Accomando et al. 2020; Bodson et al. 2007; Au and Moore 1984; Branstetter and Mercado III 2006; Holt et al. 2004) which likely combine to aid the animal in separating auditory events, thus improving detection performance.

**Fig. 17:**
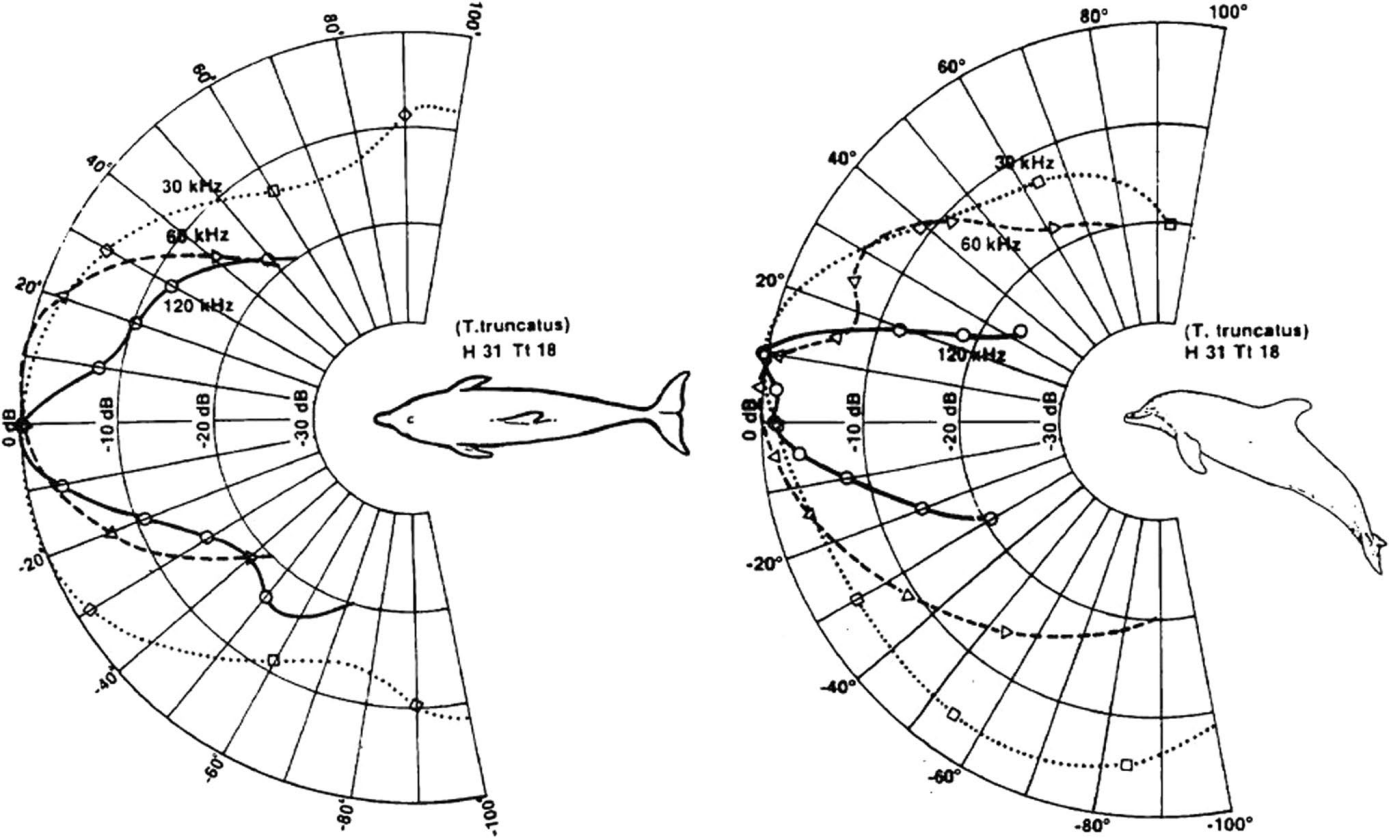
Receiver beam patterns for the bottlenose dolphin in the horizontal plane (left) and vertical plane (right) (Adapted from Au and Moore 1984)

Au and Moore (1984) measured detection thresholds for a bottlenose dolphin listening for on-axis tones in the presence of Gaussian noise, where the angular location of the noise source varied. The most masking occurred when the signal and noise were co-located. As the angle between the signal and noise increased, a release from masking occurred that became more salient with increasing frequency (Fig. 17). While testing was not conducted below 30 kHz, the trend in the data suggests that SRM would be less pronounced for low-frequency communication signals.

**Fig. 18:**
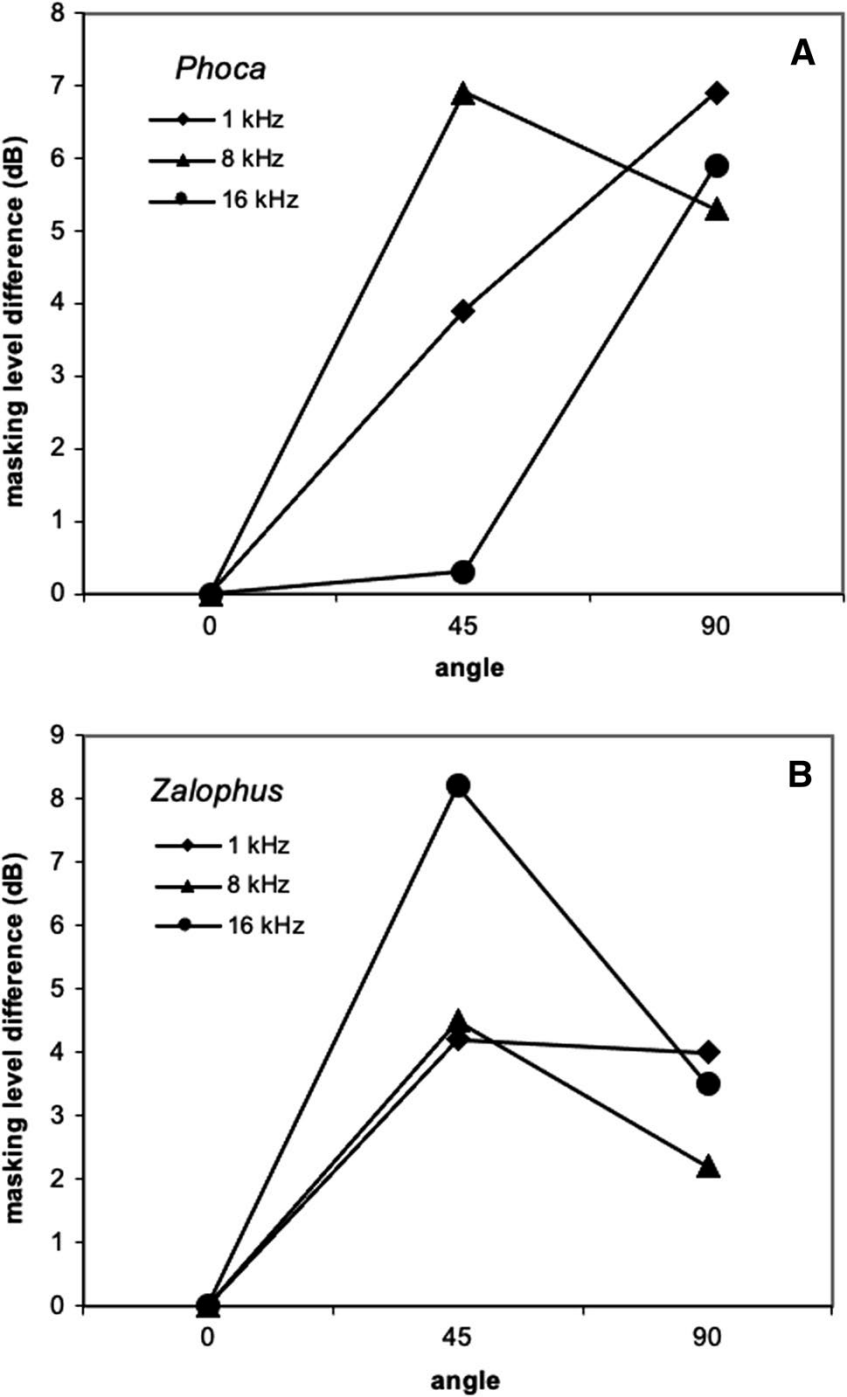
Masking level differences (in dB) relative to those measured at 0° for a harbor seal (Panel A) and California sea lion (Panel B) listening for tones projected from varying angles in the presence of on-axis masking noise. (Adapted from Holt and Schusterman 2007)

Holt and Schusterman (2007) measured SRM for airborne sounds in a harbor seal and a California sea lion. In this experiment, the location of the noise source was fixed at the on-axis position, while the location of tonal signals varied. To account for the directional sensitivity of each animal (i.e., detection thresholds vary as a function of angular location even without masking noise), the masking level difference (MLD) in dB was calculated

12 $${\text{MLD }} = \, \left( {M_{q} {-} \, M_{o} } \right) \, {-} \, \left( {U_{q} {-} \, U_{o} } \right),$$

where *U*_*o*_ and *U*_*q*_ were the unmasked detection thresholds at 0° and *q*°, respectively, and *M*_*o*_ and *M*_*q*_ were the masked detection thresholds at 0° and *q*°. The pattern for MLD was complex, but greater levels were generally measured when signal and noise were separated (Fig. 18). Overall, there was an improvement in sensitivity due to spatial separation of signal and noise sources of up to 19 dB for the harbor seal and 12 dB for the sea lion.

The effect of SRM is profound (greater than 20 dB in dolphins), especially for higher frequencies. This effect is likely due to inter-aural loudness differences resulting from sound shadowing. However, current data are limited. For the dolphin in Au and Moore (1984), the noise source was always fixed at 0° azimuth, and for the pinnipeds in Holt and Schusterman (2007), the tonal source was always fixed at 0° azimuth. Although the focus in the current paper is on behavioral studies, it is important to note that an evoked potential study did measure SRM where the locations of both the signal and noise varied on the horizontal axis (Popov et al. 2020). In this study, the signal was restricted to one frequency, a 64-kHz tone pip, and the masker was band-pass noise between 40 and 90 kHz. The most SRM occurred when the signal and noise were ipsilateral to each other; however, the effect was only 8 dB. Because sound sources are much more likely to be separated in space than co-located, more research on SRM is warranted, especially for lower frequencies similar to both communication signals and anthropogenic noise.

## Energetic vs informational masking

**Fig. 19:**
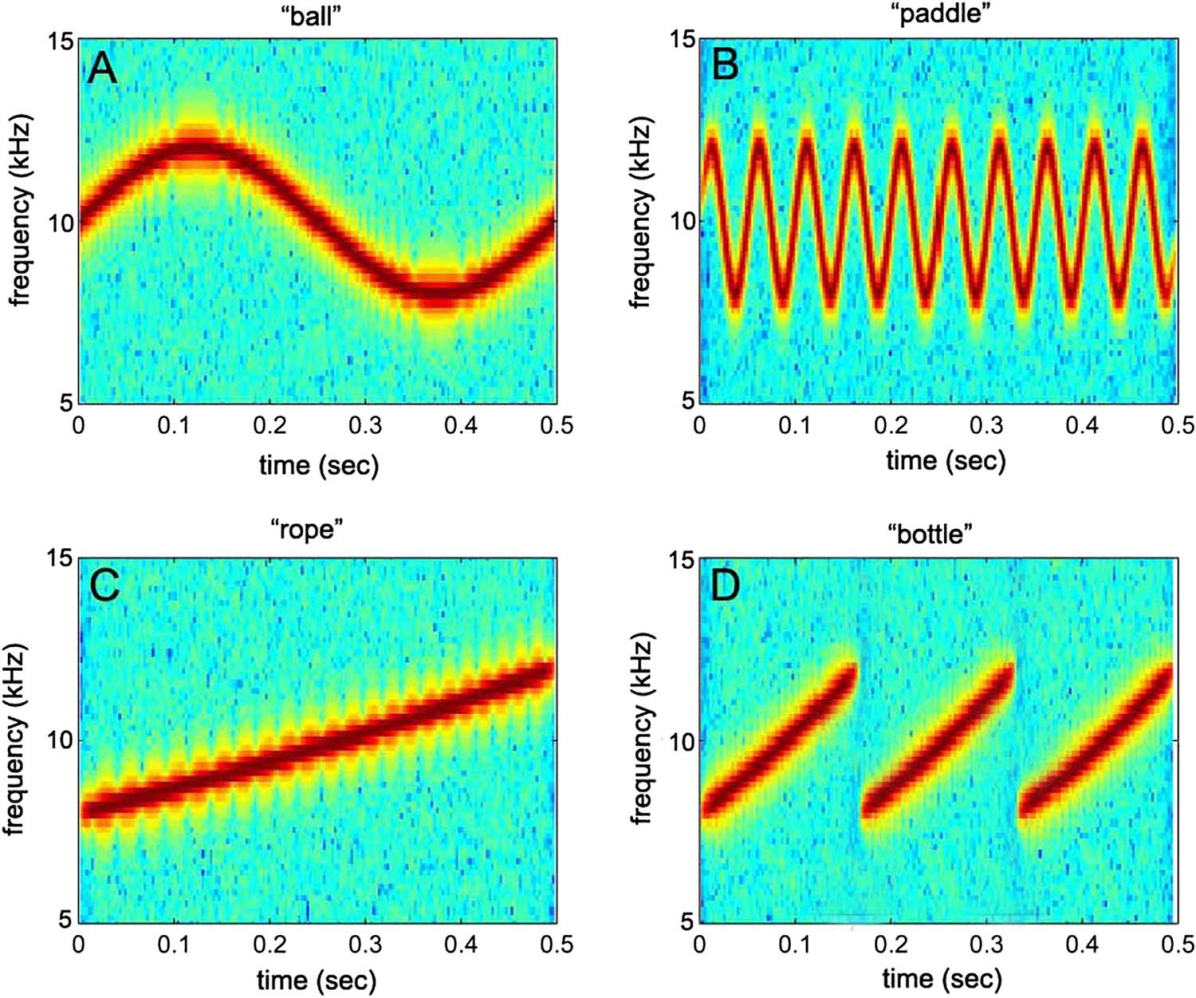
Spectrograms of FM signals. The title of each panel represents the physical object that was associated with each signal in a matching-to-sample task. (Adapted from Branstetter et al. 2016)

**Fig. 20:**
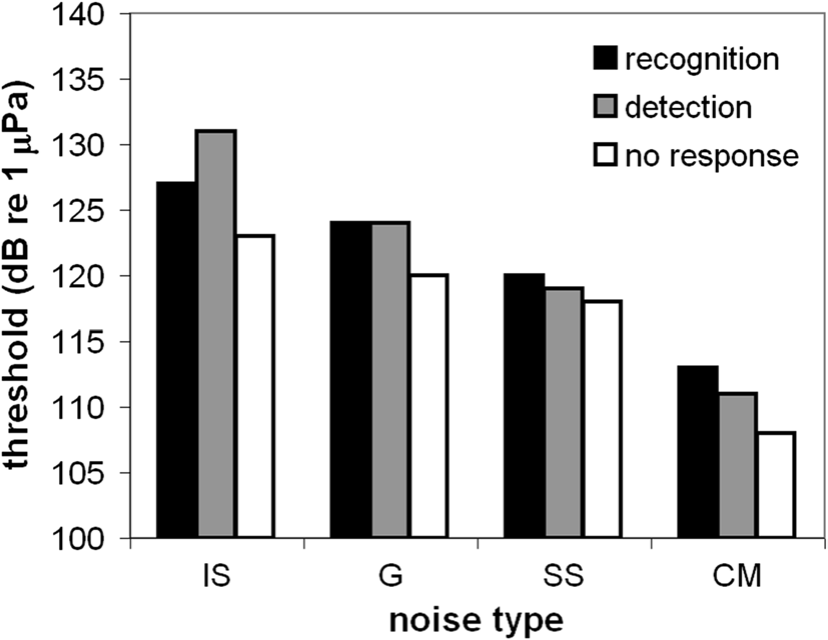
Recognition, detection, and no-response thresholds in four different noise types: ice squeaks (IS), Gaussian (G), snapping shrimp (SS), and comodulated (CM) noise. (Adapted from Branstetter et al. 2016)

The definitions of energetic and informational masking vary (Branstetter et al. 2016; Kidd et al. 2008; Pollack 1975; Wilson et al. 2012). However, many definitions suggest that energetic masking is a sensory phenomenon that occurs at the auditory periphery (e.g., Recio-Spinoso and Cooper 2013), while informational masking encompasses a wide variety of perceptual faculties including attention, memory, and recognition, and occurs at later stages of auditory processing (e.g., Branstetter et al. 2016; Kidd et al. 2008). For example, the ability to recognize human speech may be degraded in a noisy background. A human listener may clearly detect that a sound is present and even identify the sound as human speech without comprehending the semantic component of the sentence. Similarly, a bottlenose dolphin may be able to detect a whistle masked by noise, but unable to recognize the frequency contour and, thus, unable to recognize the identity of the caller. Dolphins use “signature whistles” or stereotyped whistles that uniquely identify the caller to conspecifics (Caldwell et al. 1990; Janik and Sayigh 2013). The stereotyped frequency-modulated pattern is used for recognition, while amplitude may encode other information such as emotional state (Jones et al. 2022). In an experiment in Branstetter et al. (2016), whistle-like FM signals were created to be equal in duration (500 ms) and bandwidth (8 kHz to 12 kHz) but vary in frequency contour (Fig. 19). Masked detection thresholds (energetic masking) were first measured for each signal using a standard go/no-go detection task with four noise types: ice squeaks, Gaussian, snapping shrimp and comodulated. The dolphin was then trained to associate each FM signal with a specific object using a matching-to-sample (MTS) paradigm. For example, when a linear FM sweep was presented (Fig. 19C), the dolphin was trained to swim and touch a rope. When a one-cycle sinusoidal FM signal was presented (Fig. 19A), the dolphin was taught to swim and touch a steel ball. Masked recognition thresholds were then measured using a three-alternative, forced-choice MTS test, using the same noise types from the signal detection task. Although the dolphin performed a forced-choice task where guessing would result in a correct answer 33% of the time, the dolphin did not respond at all for lower signal-to-noise ratios. As a result, no-response thresholds—defined as the average SPL where the dolphin only responded 50% of the time—were also reported. On average, recognition thresholds were 4 dB greater than no-response thresholds (Fig. 20). This result suggests that the cognitive processing needed to recognize the FM pattern and match it to a physical object required an additional 4 dB. This result is consistent with a 6-dB difference found between recognition and detection thresholds in birds (Dooling et al. 2009). Curiously, signal detection thresholds were slightly elevated relative to the no-response thresholds, even though, hypothetically, they are measuring the same thing. However, methodological differences related to the go/no-go vs the MTS tasks may have had different sustained attention demands that accounted for this difference (Branstetter et al. 2016). For example, in the MTS task, the signal was guaranteed to be presented within 5 s after the dolphin stationed on the bite plate, which is a relatively short amount of time to remain vigilant. However, for the go /no-go task, signals could occur anytime during a dive, and each dive lasted between 5 and 60 s, a much longer duration to remain vigilant. Another hypothesis is that the forced-choice task increases motivation to respond (compared to a go/no-go task) because guessing results in fish 33% of the time.

**Fig. 21:**
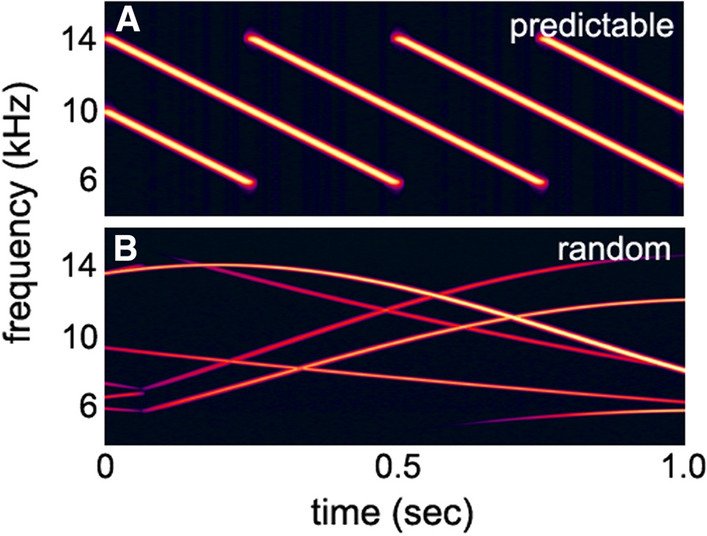
Spectrograms of two different FM noise types, one with a predictable FM pattern and one with a random frequency contour. (Adapted from Branstetter et al. 2016)

**Fig. 22:**
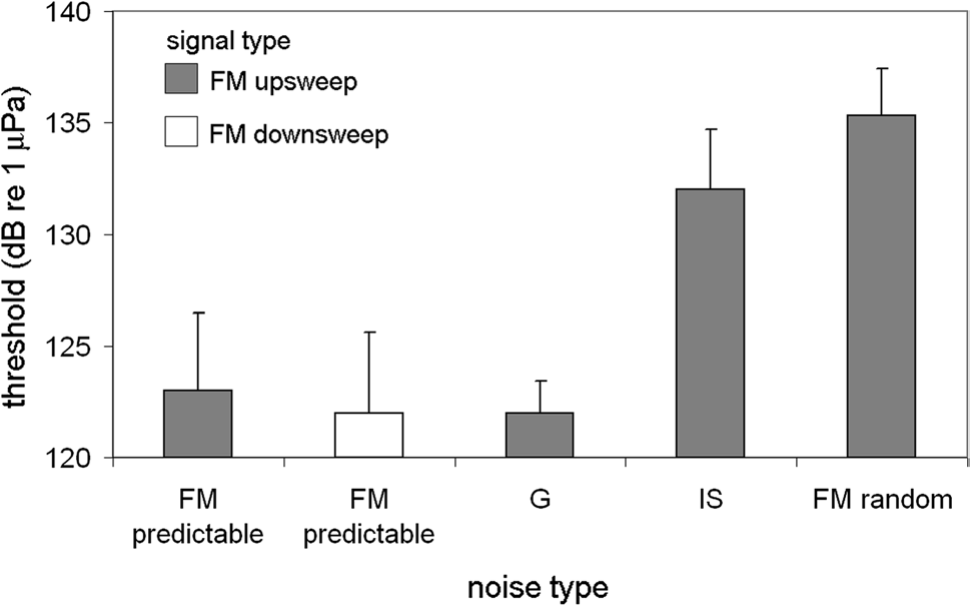
Detection thresholds for two different signals presented within four different noise types. Spectrograms of FM predictable and FM random noise are displayed in Fig. 21. G and IS are Gaussian and Ice Squeak noise, respectively. (Adapted from Branstetter et al. 2016)

Ice squeak noise consistently produced the largest masked detection and recognition thresholds in this experiment (Figs. 13 and 20). Curiously, detection thresholds in IS noise were higher than recognition thresholds, which is a non-intuitive result. However, the similarity between the FM signals and the FM ice squeaks suggests that during the detection task, the signal likely registered in the dolphin’s auditory system, but was misclassified as noise. To test this hypothesis, two FM noise types were created where one had a predictable FM pattern, a linear down sweep (Fig. 21A), and the second noise type resembled IS noise—the frequency contour was random (Fig. 21B). Both noise types were presented at the same spectral density level. Detection thresholds were measured for two 500 ms signals with bandwidths between 8 and 12 kHz: a linear upsweep and a linear down sweep. Detection thresholds for both signal types in predictable FM noise were similar to detection thresholds in Gaussian noise (Fig. 22). However, detection thresholds in random FM noise produced strikingly elevated levels of masking, even higher than IS noise. When the noise had a predictable pattern, the presentation of the signal disrupted the pattern and was easy to identify. Masking levels were consistent with the PSM and represented energetic masking. However, when the background noise was random, the presentation of the signal was wrongfully classified as part of the background noise. In this case, thresholds were elevated even though the dolphin could likely hear the signal, potentially due to informational masking. In this study with simple linear upsweeps and down sweeps, there was a 12 dB difference between apparent signal detection and signal recognition; however, the frequency contour of dolphin whistles tends to be much more complex, which could possibly allow enhanced recognition in noisy backgrounds.

## Conclusions

Energetic masking likely occurs at the level of the cochlea (Recio-Spinoso and Cooper 2013) and can be divided into within-channel processing such as that described by the power spectrum model, and across-channel processing such as that which enables comodulation masking release. Noise types that lack significant amplitude modulation are often well described by the power spectrum model. Masking by impulsive sounds, such as seismic air guns, appears to be well predicted by the PSM with the addition of a time-window analysis that considers varying noise levels within shorter temporal intervals (Sills et al. 2017). For continuous amplitude-modulated noise, a dip-listening or valley-listening strategy is most effective for relatively lower AM rates, depending on the species. As AM rates increase, temporal resolution “smears” the valleys and detection thresholds approach levels similar to Gaussian noise (Branstetter and Finneran 2008; Kastelein et al. 2021). When broadband noise is coherently amplitude modulated across auditory filters, comodulation masking release can occur. The CMR effect can be disrupted by decorrelating the noise across channels, providing strong evidence that the auditory system compares temporal envelopes across auditory filters—or possibly groups sounds with similar temporal envelope patterns across frequency regions—to enhance signal detection (Branstetter et al. 2013a; Dau and Kollmeier 1997).

Although studies of masked signal detection or energetic masking are useful for informing communication space models (Clark et al. 2009; Erbe et al. 2016; Jensen et al. 2012), animals not only need to detect a signal, they need to recognize the signal, as well. Being able to recognize a signal 80% of the time would have a much higher fitness utility than detection of a signal 50% of the time, yet 50% detection thresholds are the standard metric that inform most models of masking (Branstetter et al. 2016; Clark et al. 2009). Currently, only one marine mammal study has investigated informational masking, resulting in recognition thresholds that are approximately 4–12 dB greater than detection thresholds (Branstetter et al. 2016). The exact mechanisms responsible for informational masking are unknown. However, failure to attend to the signal, or to segregate the signal from the background due to similarity, are likely candidates. A realistic masking scenario could be investigated by measuring recognition thresholds for a complex signal (e.g., a conspecific vocalization) in naturally occurring comodulated noise (e.g., snapping shrimp), where the noise is spatially separated from the signal. This is the type of acoustic environment that many species of coastal odontocetes and pinnipeds likely experience; however, this masking scenario has not been evaluated experimentally . A significant release from masking would be predicted from the combined effects of (1) enhanced detection of the complex signal and (2) masking release from the comodulated noise and the spatially separated sound sources.

Accurate predictions of auditory masking are necessary to inform best management practices for marine mammals. Relatively simple masking models such as the power spectrum model—based on auditory filters and combined with standard critical ratios—can be applied effectively in certain situations. However, masking release (or conversely, elevated levels of masking) due to the spectral-temporal features of signals and noise and the spatial relationship of sound sources can complicate efforts to predict masking in realistic scenarios. More research is needed to better understand the mechanisms of auditory masking in marine mammals, and to improve the accuracy of masking predictions in the marine environment.
